# Cat at home? Cat scratch disease with atypical presentations and aggressive radiological findings mimicking sarcoma, a potential diagnostic pitfall

**DOI:** 10.1080/17453674.2021.1941624

**Published:** 2021-06-24

**Authors:** Florian Amerstorfer, Jasminka Igrec, Thomas Valentin, Andreas Leithner, Lukas Leitner, Mathias Glehr, Jörg Friesenbichler, Iva Brcic, Marko Bergovec

**Affiliations:** a Department of Orthopedics and Trauma, Medical University of Graz, Graz; b Division of General Radiology, Department of Radiology, Medical University of Graz, Graz; c Section of Infectious Diseases and Tropical Medicine, Department of Internal Medicine, Medical University of Graz, Graz; d Diagnostic and Research Institute of Pathology, Medical University of Graz, Neue Graz, Austria

## Abstract

Background and purpose — Cat scratch disease (CSD) is a self-limiting disease caused by *Bartonella (B.) henselae.* It is characterized by granulomatous infection, most frequently involving lymph nodes. However, it can present with atypical symptoms including musculoskeletal manifestations, posing a diagnostic challenge. We describe the prevalence and demographics of CSD cases referred to a sarcoma center, and describe the radiological, histological, and molecular findings.

Patients and methods — Our cohort comprised 10 patients, median age 27 years (12–74) with clinical and radiological findings suspicious of sarcoma.

Results — 7 cases involved the upper extremities, and 1 case each involved the axilla, groin, and knee. *B. hensela*e was found in 6 cases tested using polymerase chain reaction and serology in 5 cases. 9 cases were soft tissue lesions and 1 lesion involved the bone. 1 patient had concomitant CSD with melanoma metastasis in enlarged axillary lymph nodes. On MRI, 5 soft tissue lesions were categorized as probably inflammatory. In 3 cases, with still detectable lymph node structure and absent or initial liquefaction, the differential diagnosis included lymph node metastasis. A sarcoma diagnosis was suggested in 4 cases. The MRI imaging features of the bone lesion were suspicious of a bone tumor or osteomyelitis.

Interpretation — Atypical imaging findings cause a diagnostic challenge and the differential diagnosis includes malignant neoplasms (such as sarcoma or carcinoma metastasis) and other infections. The distinction between these possibilities is crucial for treatment and prognosis.

*Bartonella (B.) henselae* infection with regional lymphadenopathy may mimic neoplastic processes such as soft tissue or bone tumor, metastasis, or lymphoma, leading to a delayed diagnosis and unnecessary invasive procedures resulting in overtreatment (Huang et al. 1989, Gielen et al. 2003, Mazur-Melewska et al. 2015). The classical differentiation of CSD from soft tissue neoplasm are enlarged lymph node with preserved hilar architecture and reactive changes of the surrounding fat and fascia, suggesting inflammation (Wang et al. 2009, Mazur-Melewska et al. 2015, Bernard et al. 2016, Chen et al. 2018). In atypical CSD cases, soft tissue mass or a solitary bone lesion may mimic a sarcoma due to the overlapping clinical and radiological findings. Previous studies, analyzing *B. henselae* infections and their clinical and radiological presentation, are mainly focused on imaging features of lymphadenopathy/lymphadenitis at the epitrochlear region (Gielen et al. 2003, Bernard et al. 2016, Chen et al. 2018). However, the assessment regarding the potential differential diagnosis of sarcoma is scarce, as only single cases of atypical *B.henselae* infection mimicking sarcoma have been published so far (Frank et al. 1952, Nimityongskul et al. 1992, Fox and Gurtler 1993, Eichhorn-Sens et al. 2008, Colman et al. 2014, Dhal et al. 2020). To our knowledge, this retrospective study represents the first large case series of atypical CSD cases who were transferred to a sarcoma center with the primary diagnosis of sarcoma. The objectives of this study were (i) to describe the prevalence and demographics of atypical CSD cases in a musculoskeletal, orthopedic sarcoma center, (ii) to describe the radiological, histological, and molecular findings, (iii) to highlight the specific MRI criteria for differentiation, and (iv) to discuss differential diagnoses with the aim of raising awareness of this rare disease.

## Patients and methods

**Figure 1.**
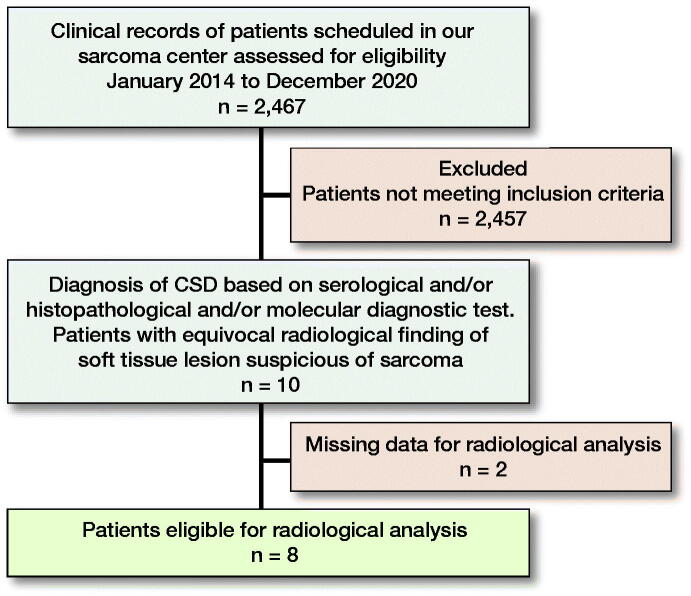
Diagram depicting the patient population and the reasons for inclusion in our cohort.

2,467 cases of soft tissue tumors (2,077 benign and 390 malignant) presented at the Sarcoma Center, Comprehensive Cancer Center, Medical University of Graz from 2014 until the end of 2020 and were searched for the terms “*B. henselae,*” “CSD,” and “granulomatous infection” originating in soft tissue, bone and/or lymph node (Figure 1). Inclusion criteria were: (1) diagnosis of CSD by either serology and/or histopathology, as well as molecular diagnostic tests and (2) clinical and/or radiological findings suspicious of soft tissue sarcoma.

The analysis included clinical data (age, sex, symptoms, diagnostic pathway, treatment, and follow-up) and MRI, histological, serological, and/or molecular findings.

MRI analyses of the lesions included: site, number of lesions, size, margin, signal intensity, and contrast enhancement (CE) pattern, changes of the surrounding fat and fascia, and differential diagnosis, and was performed by the musculoskeletal radiologist (JI) without the knowledge of the diagnosis of CSD. The study’s minimal MRI protocol included T1-weighted and a fluid-sensitive, fat-saturated (FS) sequence parallel to the lesion’s long axis, an axial proton-density, or T2-weighted FS sequence. When applicable, the postcontrast sequence after intravenous application of gadolinium (Gd) was included in the analysis.

Available hematoxylin and eosin (HE) slides, cut in 4 µm-thick whole tissue sections from formalin-fixed, paraffin-embedded (FFPE) paraffin tissue blocks, were evaluated by a bone and soft tissue pathologist (IB).

### Molecular analysis (PCR amplification of Bartonella spp. genes)

FFPE tissue blocks were cut in 30 x 5 µm-thick whole tissue sections with a microtome using a new sterile blade for each case. According to the manufacturer’s recommendations, genetic DNA was extracted with the Maxwell 16 FFPE Plus LEV DNA purification kit (Promega, Mannheim, Germany). The concentration and quality of DNA were determined spectrophotometrically with a NanoDrop ND-3300 instrument and the PicoGreen assay (Thermo Fisher Scientific, Waltham, MA, USA). As previously described (Raoult et al. 2006), the Bartonella genus-specific real-time polymerase chain reaction (PCR) amplification was performed on a LightCycler 2.0 instrument using the LightCycler® TaqMan® Master (Roche Diagnostics, Mannheim, Germany) according to the manufacturer’s recommendations with 200–750 ng input DNA. The AMPLIRUN® Bartonella DNA control (Vircell, Granada, Spain) served as a positive control.

### Ethics, funding, and potential conflicts of interests

This retrospective study (EK 30-075 ex 17/18) was approved by the Ethical Review Board. The authors have not received any funding and have no potential conflicts of interests.

## Results

### Clinical findings (Table 1)

10 cases of CSD with radiological findings suspicious of malignancy were identified, resulting in a low prevalence of 0.4% CSD cases in our musculoskeletal sarcoma center. 8 of the patients were primarily evaluated in an external institution and referred to our Center for further work-up. An additional 2 patients presented to our outpatient clinic with MRI findings suspicious of soft tissue tumor and atypical clinical presentation (growing lesions in the soft tissues of the knee and epitrochlear with unclear trauma without inflammation).

The patients’ ages ranged from 12 to 74 years (median 27 years); 6 were female. Medical history revealed swelling for several weeks prior to first presentation, median 31 days (17–270). In 7 patients the lesion was located in the upper extremities (elbow [n = 5] and upper arm [n = 2]), followed by the groin/inguinal region (n = 1), axilla (n = 1), and knee (n = 1). Pain was present in 6 cases, and in 1 case signs of infection at the middle part of the left upper arm were noted. In all cases, soft tissue neoplasms were suspected by the external radiologist or clinician. In addition, in case number 5, the lesion additionally involved bone, and chronic osteomyelitis was considered as a differential diagnosis. Only in 5 patients were cat scratches on the lower arm or hand and/or contact with cats documented. Inflammatory serum markers were documented in 8 patients, showing elevated C-reactive protein (CRP) values in only 2 cases. In contrast, normal CRP levels were documented in 6 of the cases.

### MRI imaging analysis (Table 2)

In 8 cases, MRI images were available for radiological analysis, and in 2 cases, MRI images were not available for re-evaluation.

The average diameter of the soft tissue lesions was 40 mm (21–110). The dimensions of the intra- and extraosseous extension of the bone lesion in case number 5 measured 29 x 26 x 11 mm. All soft tissue lesions were located epifascially. 2 soft tissue lesions had a well-defined margin, while the rest showed an infiltrative margin. Moreover, 2 lesions had a solid-cystic appearance and in the rest of the lesions no necrosis was observed. Additionally, in 2 lesions (cases 8 and 9) satellite lymph nodes were present.

**Figure 3.**
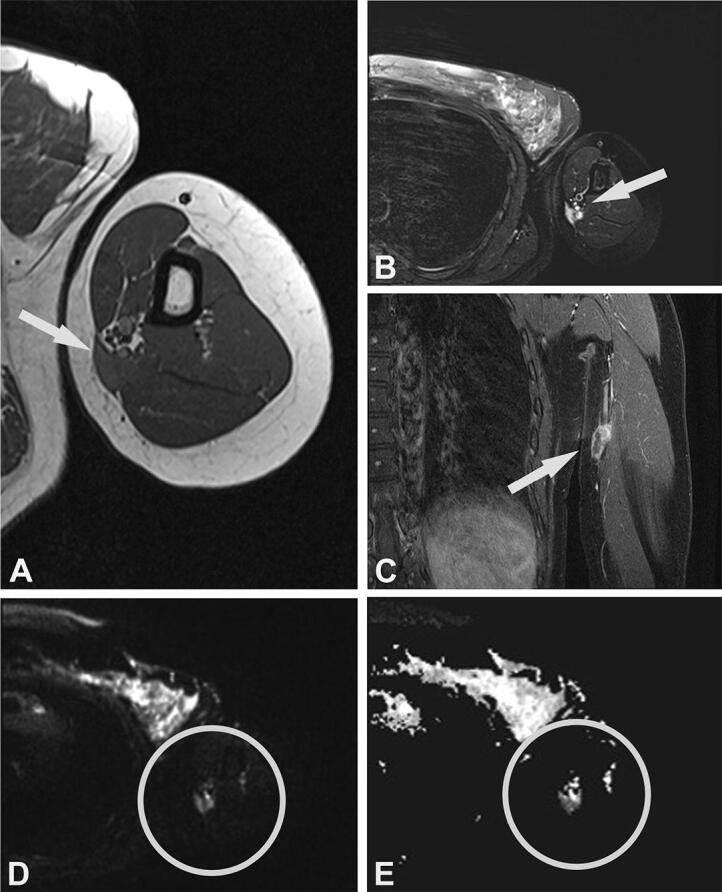
Case no. 7. A 40-year-old female with an epifascial soft tissue lesion of the upper arm. (A) On a T1-weighted image, the lesion with intermediate signal intensity and irregular margin; wide contact with the underlying fascia (arrow). (B) Homogenous high T2-weighted signal intensity without any surrounding edema (arrow). (C) Coronal T1-weighted image with fat saturation after application of Gd-contrast shows heterogeneous contrast enhancement (arrow). (D–E) DWI image (D) with corresponding (E) ADC map shows diffusion restriction due to necrotic collection (circles).

Due to MRI characteristics, 6/8 lesions were categorized as probably inflammatory (5 soft tissue lesions and 1 bone lesion). In 3 cases, with still detectable lymph node structure and absent or initial liquefaction, the differential diagnosis included lymph node metastasis. In 2 cases, nodular fasciitis was suspected with an epifascially located mass in the infrapatellar region of the knee and upper arm (Figures 2 and 3) and sarcoma was suggested in 4 cases. In comparison with radiography, the MRI imaging features of the bone lesion (Figure 4) were suspicious of a bone tumor (such as lymphoma or Ewing sarcoma) or osteomyelitis. In case number 9, atypical MRI imaging findings included metastasis in the differential diagnosis (Figure 5).

Figure 2.Case no. 10. A 37-year-old female with an infrapatellar infiltrative soft tissue lesion of the knee. (A) Proton density (PD) image shows irregular heterogeneous soft tissue lesion in subcutaneous tissue just anterior to patellar ligament with surrounding edema (white arrow) and joint effusion. (B) Homogenous hypointense signal intensity on T1-weighted image (white arrow). (C) Axial PD image shows the hyperintense lesion and extension of soft tissue edema (white arrow). (D) Sagittal T1 VIBE image after application of Gd-contrast shows homogenous contrast enhancement of the lesion with an irregular margin (white arrow). On all sequences, the patellar ligament is intact. (E–F) Histology shows fatty tissue with extensive fibrosis and numerous granulomas with central necrosis. (G–H) Central necrosis (black stars) is often stellate in appearance with admixed neutrophils and surrounded by palisading histiocytes (black arrows, H).

**Figure:**
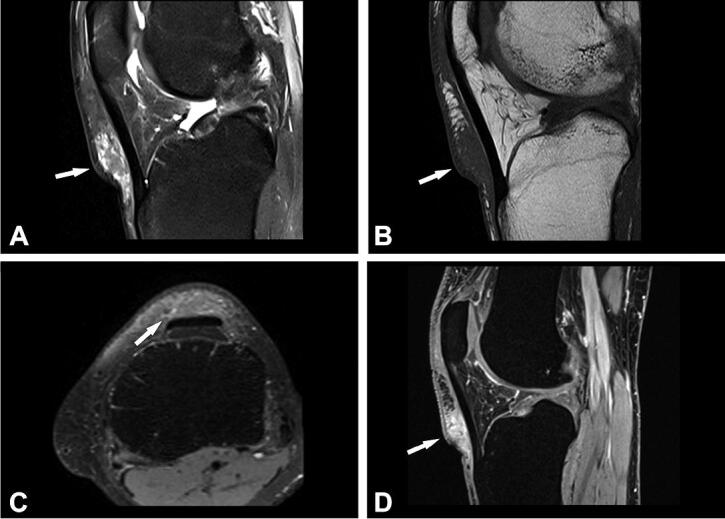

**Figure:**
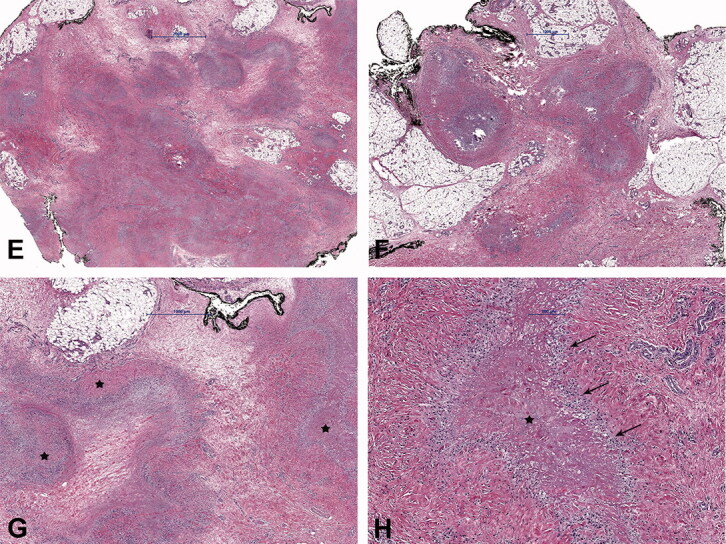

Figure 4.Case no. 5. A 13-year-old female with osteomyelitis of distal humerus as a late manifestation of cat scratch disease. (A) AP radiography of the elbow with discrete permeative osteodestruction pattern, cortical irregularity, and no periosteal reaction (circle). (B–C) Bone marrow infiltration of the medial epicondyle with permeative destruction of underlying cortex on axial proton density image (arrow, B) and coronal T1-weighted (arrow, C). (D) Bone marrow edema in the area and extraosseous extension with cortex destruction. Abnormal thickening and increased T2-weighted signal intensity within the common flexor origin from the lateral epicondyle due to inflammatory infiltration (white arrow) with edema of the surrounding subcutaneous fat tissue. (E) Corresponding color Doppler sonography shows cortical discontinuity with extraosseous soft tissue extension without significant hypervascularisation of the surrounding structures (yellow star).

**Figure:**
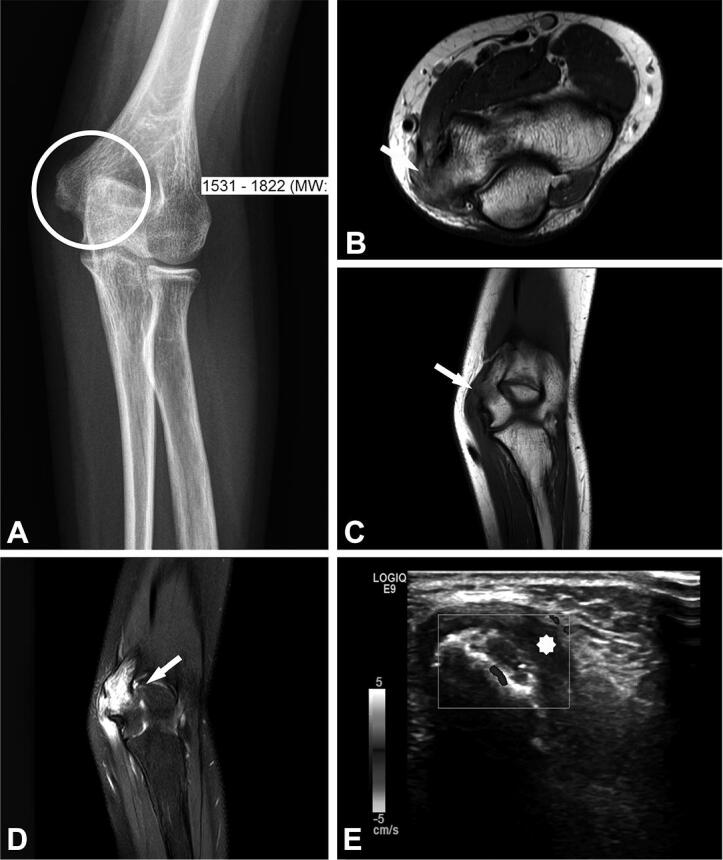

**Figure:**
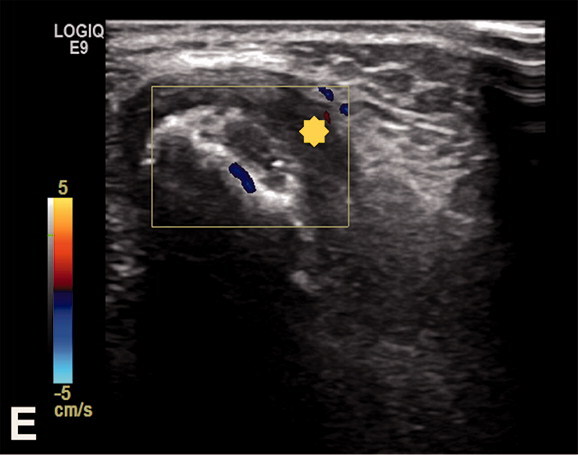

Figure 5.Case no. 9. A 74-year-old male with lymph node melanoma metastasis and synchronous cat scratch disease. (A) Enlarged axillary lymph nodes with heterogenous contrast enhancement on coronal postcontrast T1-weighted image with fat saturation; no signs of necrosis (star). (B) Sonography shows irregular 28 x 26 mm large lymph nodes with the heterogeneous structure without necrosis and hyperechogenic surrounding subcutaneous tissue (arrow). (C) On axial short tau inversion recovery (STIR) image and (D) T1-weighted image pathologically changed lymph nodes with a heterogenous signal. Centrally, on T2-weighted image low signal intensity with hyperintense signal intensity on T1-weighted image corresponds to melanin (arrows). (E) Histologically shows granuloma with central necrosis (star) and malignant melanoma metastasis (arrow). (F) Higher-power view of the granuloma with extensive central necrosis, surrounded by palisading histiocytes and (G) atypical melanocytes. (H) Malignant melanoma is immunohistochemically positive with SOX10 (Inlet: positive reaction to Melan A).

**Figure:**
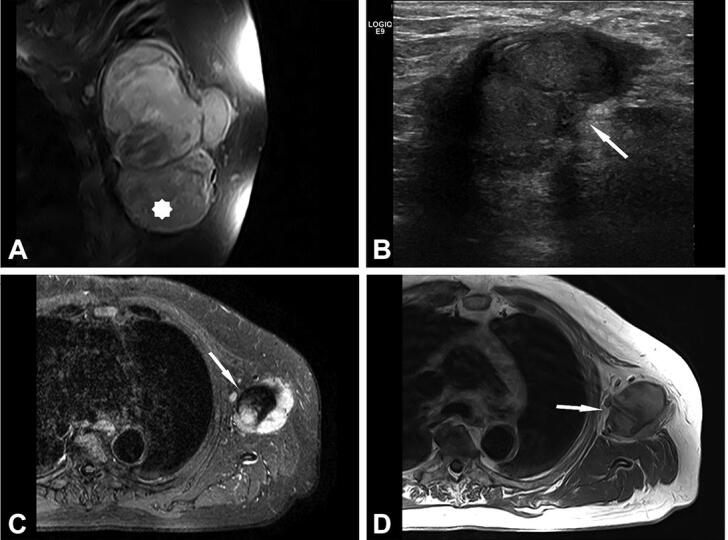

**Figure:**
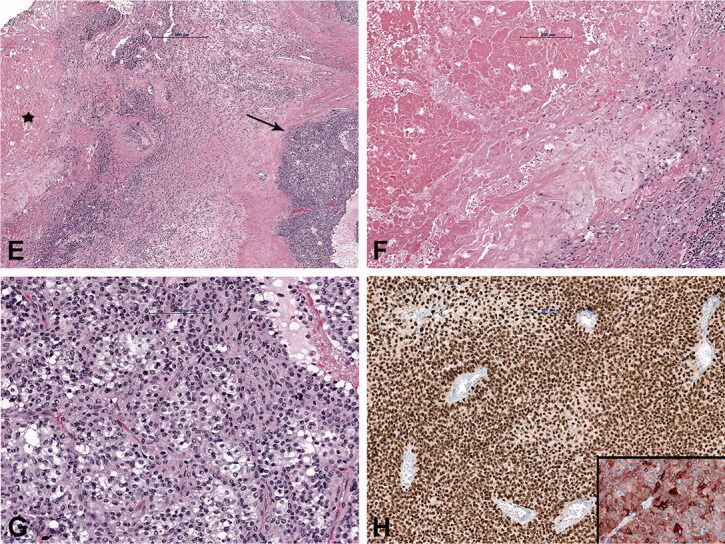

### Histological, molecular, and laboratory/serological findings (Table 1)

Due to inconclusive findings and suspicion of malignancy, core-needle, incision, or excision biopsies were performed in 6 cases. Histological examination showed numerous granulomas composed of central necrotic areas admixed with neutrophils surrounded by the palisading histiocytes (Figure 2); occasionally giant cells were also found. In 4 cases, granulomas were found only in the soft tissue and in 2 patients, soft tissues and the lymph nodes were affected. In 1 of the latter cases with soft tissue swelling in the left axilla (case number 9), a core-needle and incision biopsy was performed first, and CSD was diagnosed. However, due to findings suspicious of malignancy and after multidisciplinary discussion, an excision biopsy was performed. Histology revealed granulomatous inflammation in the soft tissue and the adjacent lymph node. A concomitant malignant melanoma composed of diffusely and atypical melanocytes arranged in clusters, with high mitotic activity and necrosis, was found in the same lymph node (Figure 5). The diagnosis was confirmed immunohistochemically by strong positive reaction of the tumor cells to S100, SOX10, HMB-45, and Melan A. To confirm the presence of Bartonella organisms, PCR analysis was performed in 6 cases and was positive. In addition, in 5 cases serologic testing for the presence of antibodies to *B. henselae* was positive. Moreover, in 5 patients, microbiological cultures were performed and all came back negative.

### Treatment and follow-up (Table 3)

In short, in 7 cases, antibiotic therapy was given for a median duration of 14 days (7–55). In the other 3 cases, patients did not receive any further therapy after the excision. Median follow-up was 45 days (11–111). In all patients, swelling and pain decreased and all were free of CSD at the last follow-up.

## Discussion

We report 10 patients with CSD, 4 of them presenting at atypical locations with soft tissue involvement mimicking sarcoma. Due to unspecific symptoms and the diversity of systemic manifestations, CSD recognition can be difficult and easily confused with soft tissue tumors. The patient’s history, physical examination, and diagnostic imaging play an important part in establishing the correct diagnosis. This helps in the prevention of further invasive procedures and the provision of rapid, appropriate treatment. 5 patients presented with visible cat scratches most frequently located on the upper extremities in our case series, as previously published (Carithers 1985, Gielen et al. 2003, Raoult et al. 2006).

Sonography, an initial imaging method used for the assessment of superficial soft tissue tumors, is used also in typical CSD, demonstrating enlarged hypovascularized lymph node(s) adjacent to the entry point of infection with different degrees of liquefaction (Mazur-Melewska et al. 2015, Bernard et al. 2016). On MRI, in early disease, the affected enlarged lymph node has a typical appearance with a thickened cortex and timely evolution of the sharp delineated perinodal abscesses with gradual necrosis of the lymph node (Mazur-Melewska et al. 2015, Chen et al. 2018). Computed tomography (CT) is inferior to MRI because of its limited differentiation of soft-tissue structures and edema (Bernard et al. 2016).

An atypical manifestation of CSD, such as knee soft tissue mass and osteomyelitis, as in our cohort, is rare and mainly affects children (Carithers 1985, Robson et al. 1999, Donà et al. 2018). Osteomyelitis associated with *B. henselae* frequently occurs along with regional lymphadenopathy due to hematogenous or lymphatic spread after the inoculation of the microorganisms (Florin et al. 2008, Erdem et al. 2018). In atypical CSD, MRI is the imaging technique of choice in characterization of inflammatory soft tissue mass with adjacent soft tissue edema and in evaluation of bone lesions (Mazur-Melewska et al. 2015, Erdem et al. 2018). For bone lesions, radiography or CT are complementary imaging methods to MRI, useful in evaluating the bone destruction pattern and periosteal reaction. A permeative destruction pattern with cortical destruction and extraosseous soft tissue extension can mimic primary malignant bone processes, as seen in one of our patients (Figure 4, case number 5).

Histologically, the finding of granulomatous inflammation with central necrosis is a feature associated with various infections, such as CSD and tuberculosis caused by Mycobacterium spp. Therefore, further analysis of the acquired specimens should be performed. Due to its slow growth, *B. henselae* is difficult to culture. Better diagnostic methods for detecting *B. henselae* include serologic testing and molecular analysis, such as PCR amplification of Bartonella spp. genes (Sander et al. 1998, 1999, Herremans et al. 2007, Edouard et al. 2015). In our study, in all 6 patients tested prior to antibiotic therapy, PCR was positive and in 5 patients, serology for *B. henselae* came back positive. Of note, a positive *B. henselae* serology or PCR does not exclude other etiology, such as malignancy or osteomyelitis (Florin et al. 2008). In the study by Rolain et al. (2006), the histopathological analysis of 181 lymph node biopsy specimens from patients with suspected CSD showed concurrent diseases in 13 *B. henselae* positive patients: 10 patients had a mycobacterial infection, and 3 had a malignant disease (2 lymphomas and 1 Hodgkin disease). Biopsy is not indicated in lesions with unequivocal clinical and imaging features of inflammation. In atypical cases, when *B. henselae* PCR is positive and histology characteristic, concurrent malignancy (primary or metastasis) should be ruled out by performing a biopsy (Florin et al. 2008, Baranowski and Huang 2020). In our study, in 3 patients biopsy was performed, as radiological findings were suspicious of malignancy despite positive serology in 1 of the cases, and in 1 patient a synchronous metastasis of malignant melanoma was found.

Treatment of CSD frequently includes surgery and antibiotic therapy. Up to one-third of cases develop suppuration of the affected lymph nodes and evacuation of the pus is indicated (Wang et al. 2009). Lymphadenectomy is not advised due to the possibility of the development of fistulas (Baranowski and Huang 2020). In our study, even though *B. henselae* infection was confirmed in 2 cases, lymphadenectomy was performed due to imaging findings suspicious of malignancy. 1 patient (case number 4) was free of local postoperative complications and in the second patient (case number 9) histology showed granulomatous inflammation with synchronous metastasis of the malignant melanoma. This case illustrates that only histologic analysis can rule out malignant diseases, and if the findings are inconclusive, the biopsy should be repeated.

The radiological differential diagnosis of CSD includes other infections and a range of benign and malignant soft tissue tumors, such as peripheral nerve sheath tumors, synovial sarcoma, leiomyosarcoma, and distant nodal metastasis (Mazur-Melewska et al. 2015, Bernard et al. 2016, Chen et al. 2018). As seen in our study, the radiologist’s experience and knowledge of different imaging features may play an important role in diagnosing typical and atypical findings in the spectrum of CSD, resulting in a significantly lower number of equivocal findings suspicious of malignancy. In addition, for the differentiation of lymph nodes in the epitrochlear region from other soft tissue masses, key anatomical features, including location posterior to the basilic vein, superficial to the brachialis muscle and brachial fascia or intermuscular septum covering the medial head of the triceps and ulnar nerve, can be used (Bernard et al. 2016).

In conclusion, this study showed a very low prevalence of 0.4% CSD cases in a musculoskeletal orthopedic sarcoma center. Radiologically, CSD is typically presented as a pathologically enlarged lymph node with different grades of liquefaction and inflammatory changes of surrounding tissues. However, in atypical cases, CSD may mimic soft tissue or bone sarcoma due to undefined soft tissue mass without discernible lymph node structure or bone involvement. These cases cause diagnostic challenges, and the differential diagnosis includes benign and malignant neoplasms (such as sarcoma or carcinoma metastasis) and infections. Besides the clinical picture, patient history, and radiologic imaging, histopathology together with PCR and serology remain the diagnostic methods of choice to establish the correct diagnosis. The distinction between these possibilities is crucial for treatment and prognosis. Furthermore, this study highlights the importance of treating patients with suspicious soft tissue lesions irrespective of size and patients with a deep-seated lesion larger than 5 cm in a specialized multidisciplinary sarcoma center.

AF, IJ, BI, BM, and FJ substantial contributed to research design, AF, IJ, BI, VT, LL, and GM analyzed and interpreted the data. AF, IJ, BI, and LA drafted the paper and all authors revised it critically. All authors have read and approved the final submitted manuscript 

*Acta* thanks Rudolf W. Poolman help with peer review of this study.

**Table 1.** Summary of clinical, serological, pathological and molecular data

Case no.	Sex	Age	Swelling (days)	CRP (< 5 mg/L)	WBC count (< 11.3 G/L)	Biopsy	Histology	Serologic testing	PCR	Culture
1	m	26	38	0.6	4.8	IB	Granulomatous inflammation with necrosis	n.a.	pos	neg
2	m	16	29	21.9	11.2	n.d.	n.a.	1:256	n.a.	n.a.
3	f	22	28	0.6	6.6	n.d.	n.a.	1:64	n.a.	n.a.
4	m	36	42	2.1	5.1	EB	Granulomatous inflammation with necrosis	n.a.	pos	neg
5	f	12	23	1.0	7.6	n.d.	n.a.	1:256	n.a.	n.a.
6	f	26	17	1.1	6.3	CNB	Granulomatous inflammation with necrosis	n.a.	pos	neg
7	f	40	33	n.a.	n.a.	n.d.	n.a.	1:64	n.a.	n.a.
8	f	27	21	16.9	7.7	EB	Granulomatous inflammation with necrosis	1:256	pos	neg
9	m	74	150	3.6	6.2	CNB,	Granulomatous inflammation with necrosis,	n.a.	pos	neg
						IB, EB	metastasis of malignant melanoma			
10	f	37	270	n.a.	n.a.	EB	Granulomatous inflammation with necrosis	n.a.	pos	n.a.

Legend: CNB: core-needle biopsy; CRP: C-reactive protein; EB: excision biopsy; IB: incision biopsy; n.a.: not available; n.d.: not done; neg: negative; PCR: polymerase chain reaction; pos: positive; WBC: white blood cell.

**Table 2.** MRI characteristics of the lesions with differential diagnosis

No.	A	B	C	D	E	F	G	H	I	J	K	L	M
1	n.a.	n.a.	n.a.	n.a.	n.a.	n.a.	n.a.	n.a.	n.a.	n.a.	n.a.	n.a.	n.a.
2	Distal UA (ST)	epi	28x45x28	c-s	inf	im	h	yes	rim	yes	no	Inflammation	Vascular lesion with internal bleeding
3	n.a.	n.a.	n.a.	n.a.	n.a.	n.a.	n.a.	n.a.	n.a.	n.a.	n.a.	n.a.	n.a.
4	Inguinal (ST)	epi	29x15x35	s	wd	h	h	no	hetero	yes	no	Pathologic LN	Soft tissue tumor (synovial sarcoma, neural tumor)
5	Distal UA (ST + B)	no	29x26x11	s	inf	im	h	yes	no	no	no	Inflammation; bone sarcoma	Osteomyelitis; bone tumor (Ewing sarcoma)
6	Distal UA (ST)	epi	26x14x24	s	inf	h	h	yes	hetero	yes	no	Inflammation; STS	venous structure; hematoma
7	Proximal UA (ST)	epi	18x6x21	s	inf	im	h	yes	rim	yes	no	Inflammation; nodular fasciitis; STS	Sarcoma
8	Proximal LA (ST)	epi	37x16x22	c-s	inf	im	h	yes	hetero	yes	yes	Pathologic LN; inflammation	Sarcoma/pathologic LN
9	Axilla (ST + LN)	epi	49x75x110	s	wd	h	h	no	hetero	yes	yes	Pathologic LN; STS	Liposarcoma
10.	Knee (ST)	epi	24x25x10	s	inf	im	h	yes	homo	no	no	Nodular fasciitis; inflammation, STS	Unclear lesion

A.Location (tissue type): B: bone; LA: lower arm; UA: upper arm, ST: soft tissue; LN: lymph node

B.Fascial relation: epi: epifascial

C.Size (mm)

D.Structure: c-s: Cystic-solid; s: Solid

E.Margin:inf: infiltrative; wd: well defined

F.T1-weighted sequence, h: high; im: intermediate

G.T2-weighted sequence, h: high

H.Surrounding edema

I.Enhancement hetero: heterogenous; homo: homogenous;

J.Necrosis

K.Satellite lesion(s)

L.Differential diagnosis (internal); LN: lymph node, STS: soft tissue sarcoma

M.Differential diagnosis (external): LN: lymph node, STS: soft tissue sarcoma;

n.a.: not available;

**Table 3.** Summary of treatment and follow-up

No.	Antibiotics	Treatment length (days)	Follow-up (days)
1	ciprofloxacin/doxycycline	55	60
2	clarithromycin	7	37
3	ciprofloxacin/doxycycline	11	11
4	none	−	59
5	clarithromycin	49	100
6	doxycycline	14	33
7	azithromycin	14	111
8	none	−	11
9	doxycycline + rifampicin	28	53
10	none	−	28
